# Supplementary material for: Transcriptome Analysis Reveals Vimentin-Induced Disruption of Cell–Cell Associations Augments Breast Cancer Cell Migration
Source: Cells. 2022 Dec 13;11(24):4035. doi: 10.3390/cells11244035 (PMC9776984; doi:10.3390/cells11244035)
Supplement: Supplementary file 1 [file cells-11-04035-s001.zip › DEGs and enrichment analysis.zip!/DEGs and enrichment analysis/WT_VIMvsVector KEGG/UP/WT_VIMvsVector_UP.pathway_map.zip!/WT_VIMvsVector_UP.pathway_map/src/hsa04120.html]

hsa04120


- hsa:140739

- Up regulated genes

ENSG00000224080(2.57501965901476)

- hsa:991

- Up regulated genes

ENSG00000117399(2.41242866521263)

- hsa:27338

- Up regulated genes

ENSG00000224126(3.56654818492803) ENSG00000108106(1.81357722771686)

Close
